# Comparative analysis of intestinal flora at different overwintering periods in wild relict gulls (*Larus relictus*): first evidence from Northern China

**DOI:** 10.3389/frmbi.2023.1218281

**Published:** 2023-10-02

**Authors:** Hongyu Yao, Zeming Zhang, Nan Wu, Mengping Wang, Qian Wu, Hong Wu, Dapeng Zhao

**Affiliations:** Tianjin Key Laboratory of Conservation and Utilization of Animal Diversity, College of Life Sciences, Tianjin Normal University, Tianjin, China

**Keywords:** relict gulls, gut microbiome, high-throughput sequencing, migratory bird, bacterial composition

## Abstract

The migratory bird’s gut microbiome composition and function change during the overwintering period, helping the host to adapt to different environments. Our study investigated the gut microbiome of migratory relict gulls (*Larus relictus*) in the early and late wintering stages from their overwintering grounds in Tianjin, China. We collected 24 and 29 fecal samples at the early and late stages, respectively, and analyzed the samples using high-throughput sequencing technology to find the relationship between diet, living environment, and gut microbiome of migratory birds. The results showed that the diversity and abundance of microbial communities (alpha diversity) increased during the overwintering period and significantly differed between both groups (beta diversity). Based on the gut microbial taxonomic composition, the relative abundance of Firmicutes decreased during the overwintering period, and Proteobacteria increased significantly. Furthermore, *Catellicoccus* and *Breznakia* were the main genera in both the early and late stages. Prediction of KEGG functions based on the PICRUSt2 method showed that changes in the gut microbiome resulted in an increased abundance of bacteria associated with amino acid metabolism, metabolism of cofactors and vitamins, energy metabolism, and environmental adaptation in the late stage. Differences in diet and environment at different stages during the overwintering period may have led to the differentiation of microbial communities, and their adaptive mechanisms need to be further investigated. This is the first in-depth study of the gut microbial composition of *L. relictus* during the overwintering period in northern China. These findings may contribute to the protection of migratory birds.

## 1 Introduction

Avian species play an essential ecological function in natural ecosystems, and the study of their gut microbiomes will help us understand birds’ evolutionary biology, contributing to the protection of endangered species and their living environment (Hird, 2017; Grond et al., 2018; Ingala et al., 2018). Numerous studies have shown that habitat conditions and diet could affect the gut microbial community of wild animals (Davidson et al., 2020), and the intestinal microbiome may also change to help the host adapt to different environments (Youngblut et al., 2019). Currently, abundant studies have shown that migratory bird feces can increase the carbon, nitrogen, and phosphorus content in wetland soil, which helps maintain the diversity of wetland vegetation (Irick et al., 2015; Wu et al., 2018).

Previous studies have shown that most of the world’s birds are migratory (Grond et al., 2018), and monitoring the health of migratory birds at stopover, breeding, and wintering sites is important for the conservation of the species concerned, especially at wintering sites, which are directly related to the survival of their populations (Zhao et al., 2021). However, due to human activities and other reasons, the wintering sites of some bird species show a shrinking trend or fragmentation characteristics (Song et al., 2014). Therefore, focusing on the intestinal flora characteristics of migratory birds, especially for the wintering period of rare and endangered birds, has positive implications for the conservation of this species. Studies have illustrated that the diet and living environments directly affect the gut microbiome composition of birds (Jiang et al., 2020; Wang et al., 2021), especially migratory birds whose living conditions change during the different life activity cycles (Zhao et al., 2021). For example, Zhou et al. (2016) found significant differences between the gut microbiome communities of Tibetan chickens from five different habitats (Zhou et al., 2016). Furthermore, Jarma et al. (2021) analyzed the bacterial community of migratory waterbirds and found that the intestinal microbiome of these birds varied according to changes in feeding habits (Jarma et al., 2021). Zhao et al. (2021) elucidated the gut microbiome of black-necked cranes (*Grus nigricollis*) between early and late wintering periods, understanding the adaption mechanism of migratory birds (Zhao et al., 2021).

The relict gull (*Larus relictus*), a migratory bird, is classified as “vulnerable” in the International Union for Conservation of Nature Red List (BirdLife International, 2017; Yang et al., 2021; Wang et al., 2022). Their main breeding grounds are the Russian Far East, eastern Mongolia, and Kazakhstan; every winter, the gulls migrate from colder places to spend winter in China (Liu et al., 2017). Most individual wintering sites of the relict gulls are located in the coastal mudflats of Bohai Bay, China (Liu et al., 2017), but Tianjin Relict Gull Park is one of their main wintering sites in Tianjin, China. During migration, wild birds can obtain food from different environments and interact with other birds (Pekarsky et al., 2021). Therefore, their potential to transmit microorganisms is high (Araujo et al., 2014), and monitoring their health levels will not only help conserve endangered migratory birds but also in the timely detection of the transmission of pathogenic microorganisms. Thus, studying microorganisms from fecal samples will provide a tool to monitor potential pathogens (Turjeman et al., 2020), and changes during the overwintering period will lay the foundation for analyzing the adaptation mechanisms of relict gulls to the living environment (Sun et al., 2022). Numerous studies have shown that the dynamic adjustment of the gut microbiome composition allows the host to absorb food more efficiently to meet their energy and nutrient requirements (Hooper et al., 2002; Koren et al., 2012; Chevalier et al., 2015). However, previous studies of relict gulls have only compared gut microbiome composition at different breeding sites in Inner Mongolia (Liu et al., 2022), and comparative data on gut microbiome at early and late wintering stages have not been studied.

In this study, we for the first time identified the composition and structure of gut microbiome of relict gulls in the early and late wintering stages from Tianjin city, China, using 16S rRNA high-throughput sequencing. Furthermore, the effects of the gut microbiome on host function were characterized by PICRUST 2 at each overwintering period (early and late). These findings may provide new insight into the gut microbiome composition of *L relictus* during the overwintering period and contribute to protecting migratory birds during this time period.

## 2 Materials and methods

### 2.1 Ethics statement

The current study was based on non-invasive techniques for collecting stool samples. Since there was no direct contact with the wild animals, approval from the Institutional Animal Care and Use Committee was not required. Tianjin Normal University approved the project, and the management department of Tianjin Relict Gull Park approved the collection of fecal samples of relict gulls.

### 2.2 Sample collection

The migratory relict gull typically arrives at Tianjin Relict Gull Park in October and leaves the following Spring. Fresh feces of *L. relictus* were collected from October 2021 to January 2022 by non-invasive methods. The sampling process overlapped with the monitoring of wintering birds, and all fresh feces were collected as soon as possible after the birds had migrated under binocular observation.

A total of 53 fecal samples from multiple individuals (whole stage, named as “total group”) were collected in this study, including 24 samples collected during October 2021 (early stage, named as “E group”) and 29 samples collected during January 2022 (late stage, named as “L group”) (**Table 1**). All the samples were placed into sterilized 5 ml EP tubes using a sterilized spoon and frozen temporarily on dry ice in a portable storage box. The feces samples were immediately transported back to the laboratory and stored at -80°C before DNA extraction. The fecal samples were soaked fully in a saturated bioenzyme washing solution for 24 h, and then after washing out, food residue in feces was observed and identified with a stereo microscope.

**Table 1:** *Larus relictus* fecal sample collection information.

Sample	Collection time	Location	Vegetation of the samples
**E01-E24**	2021.10	117.80273839, 39.13956585	Grain, plants, or seeds on the migration route
**L01-L29**	2022.1	117.80273839, 39.13956585	Phytoplankton on the beach

### 2.3 DNA extraction, amplification, and sequencing of fecal microorganisms

Total fecal DNA was extracted using the TIANamp Stool DNA Kit (TIANGEN, Beijing, China), following the manufacturer’s instructions. The absorbance ratios at A260/A230 and A260/A280 were measured using a NanoDrop 2000 spectrophotometer (ThermoFisher Scientific, Wilmington, DE, USA) to assess the quality of the DNA. The DNA concentration in the final extract was measured using a Qubit 2.0 Fluorometer (Life Technologies, Carlsbad, CA, USA).

After extraction, PCR amplification of the 16S rRNA gene (V3-V4 hypervariable region) was conducted using the primers 338F 5′-ACTCCTACGGGAGGCAGCA-3′ and 806R 5′-GGACTACHVGGGTWTCTAAT-3′ (Fadrosh et al., 2014). PCR products were detected by gel electrophoresis (2% agarose gel [w/v]) and recovered by an AxyPrep DNA Gel Recovery Kit (AXYGEN Corporation, Silicon Valley, CA, USA). The concentration and purity were determined by the QuantiFluor-ST™ Blue Fluorescence Quantification System (Promega, Madison, WI, USA). The purified PCR amplicons were then sequenced on an Illumina MiSeq platform at Shanghai Majorbio Bio-pharm Technology Co., Ltd.

### 2.4 Sequence analysis

The raw data were quality-filtered and merged using QIIME 2 (Chen et al., 2018). The quality-filtered Operational Taxonomic Unit (OTUs) were taxonomically annotated by a pretrained Naive Bayes classifier based on the SILVA 138 database. ADONIS was implemented in the R vegan package, and UniFrac distances were calculated in the Mothur software (Vikram et al., 2021). A one-way ANOVA was used to assess significant differences (*p* < 0.05 was considered significant) in bacterial abundances between the early and late groups. Venn diagrams are presented to demonstrate the unique and shared OTUs. The Wilcoxon rank-sum test was used to detect variability in community abundances between different subpopulations at the phylum and genus levels with SPSS 26.0 software.

### 2.5 Statistical analyses and functional predictions

Alpha diversity indices (including the ACE, Chao1, Shannon, Simpson, and Coverage indices) were calculated using the Mothur software (v.1.30.1) to compare the richness and diversity of the microbial communities of the samples. In this study, the Wilcoxon rank-sum test was used to compare the alpha diversity parameters of species at the OTU level. The similarity of the gut microbiome of the two different groups was detected by principal coordinate analysis (PCoA) based on weighted and unweighted UniFrac metrics. PICRUSt 2 (v.2.4.1) was used to predict the 16S rRNA gene sequence and abundance based on the bacterial functional potential of the community to analyze metabolic pathways (Douglas et al., 2020).

## 3 Results

### 3.1 Sequencing data

Following Illumina MiSeq sequencing and denoising of the relict gull fecal samples, a total of 2,777,539 optimized sequences were obtained with an average length of 422 bp from 53 samples (**Supplementary Table 1**). After OTU clustering at 97% similarity, 4182 OTUs were obtained. OTUs were classified into 50 phyla, 134 classes, 323 orders, 532 families, and 1092 genera. The rarefaction based on OTU abundance reached a plateau in both the early and late groups, indicating that the amount of sequencing data was reasonable to reflect the maximum level of microbial diversity, and the species distribution was uniform (**Supplementary Figure 1**).

Among the 4182 OTUs, 1924 (46%) were shared by relict gulls between the two groups, 763 were present only in the gut microbiome of the E group, and 1495 were present only in the L group (**Supplementary Figure 1**). The number of phyla and genera shared between the two groups was 38 and 664, respectively (**Supplementary Figure 1**). The shared phyla belonged to Firmicutes (64.06%), Proteobacteria (21.73%), and Actinobacteriota (4.25%).

### 3.2 Gut microbiome taxonomic composition

**Figure 1:**
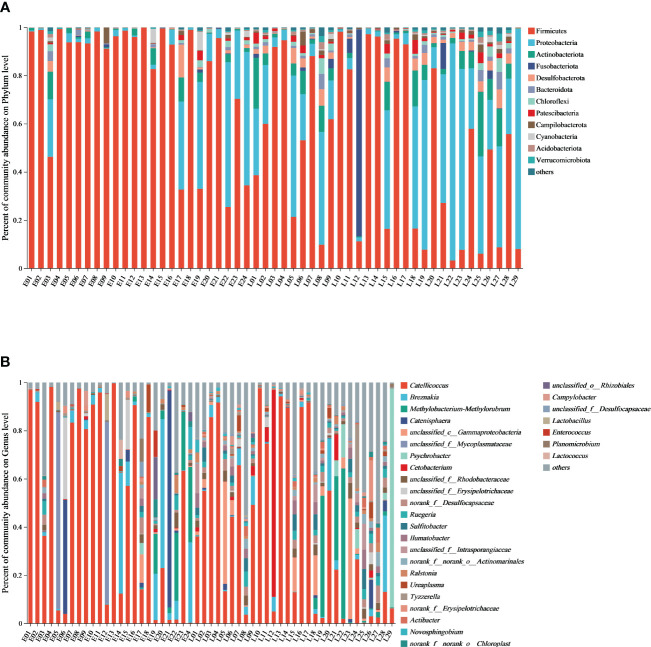
The gut microbiome composition from the relict gull at the phylum/genus level. **(A)** Microbial structure of fecal samples from the E and L group at the phylum level and **(B)** microbial structure of fecal samples from the E and L group at the genus level.

Phyla/genera with more than 1% abundance were classified as dominant phyla/genera. Based on 16S rRNA sequencing at the phylum level, Firmicutes (64.06%), Proteobacteria (21.73%), Actinobacteriota (4.25%), Fusobacteriota (2.26%), Desulfobacterota (1.92%), Bacteroidota (1.01%), and Chloroflexi (1.01%) were the dominant phyla (**Figure 1A**; **Supplementary Figure 2A**). Three and nine dominant phyla were detected for the E and L groups, respectively, with the top three for both groups being similar.

A total of 41 phyla were identified in the E group microbiome composition, with the dominant being Firmicutes (81.47%) and Proteobacteria (11.97%), followed by Actinobacteriota (2.07%). The cumulative proportion of these three phyla was above 96%. 47 phyla were identified for the L group microbiome composition, which was dominated by Firmicutes (49.64%) and Proteobacteria (29.81%), followed by Actinobacteriota (6.06%), Fusobacteriota (3.99%), Desulfobacterota (2.68%), Bacteroidota (1.45%), Patescibacteria (1.40%), Chloroflexi (1.37%) and Campilobacterota (1.07%) (**Figure 1A**; **Supplementary Figure 2A**; **Table 2**).

**Table 2:** The mean relative abundance of the top 10 most abundant at phyla level.

Sample group	Top ten abundant phyla (%)
Early (E group)	Firmicutes (81.47)
	Proteobacteria (11.96)
	Actinobacteriota (2.08)
	Desulfobacteriota (0.99)
	Cyanobacteria (0.80)
	Chloroflexi (0.57)
	Campilobacterota (0.50)
	Bacteroidota (0.49)
	Patescibacteria (0.40)
	Fusobacteriota (0.18)
Late (L group)	Firmicutes (49.64)
	Proteobacteria (29.81)
	Actinobacteriota (6.06)
	Fusobacteriota (3.99)
	Desulfobacteriota (2.68)
	Bacteroidota (1.45)
	Patescibacteria (1.40)
	Chloroflexi (1.37)
	Campilobacterota (1.07)
	Acidobacteriota (0.66)

The gut microbiome composition of the relict gulls was also analyzed at the genus level. A total of 14 bacterial genera were found, showing an average relative abundance above 1% for all samples. The dominant genera in the E group were *Catellicoccus* (51.60%), *Breznakia* (8.75%), *unclassified_f_Mycoplasmataceae* (6.61%), *Catenisphaera* (6.07%), *Methylobacterium-Methylorubrum* (4.55%) and *unclassified_f_Erysipelotrichaceae* (2.34%). The dominant genera in the L group were *Catellicoccus* (39.17%), *Methylobacterium-Methylorubrum* (5.47%), *unclassified_c_Gammaproteobacteria* (5.21%), *Psychrobacter* (4.23%), *Cetobacterium* (3.96%), *Breznakia* (3.64%), *Sulfitobacter* (1.96%), norank_f_ *Desulfocapsaceae* (1.93%), *Ilumatobacter* (1.90%), *unclassified_Rhodobacteraceae* (1.83%), *Ruegeria* (1.69%), *unclassified_Intrasporangiaceae* (1.55%), *Actinomarinales* (1.25%), *Tyzzerella* (1.13%), and *Actibacter* (1.04%) (**Figure 1B**; **Supplementary Figure 2B**; **Table 3**).

**Table 3:** The mean relative abundance of the top 10 most abundant at genus level.

Sample group	Top ten abundant gerna (%)
Early (E group)	*Catellicoccus* (51.60)
	*Breznakia* (8.75)
	*unclassified_f:Mycoplasmataceae* (6.61)
	*Catenisphaera* (6.07)
	*Methylobacterium-Methylorubrum* (4.55)
	*unclassified_f:Erysipelotrichaceae* (2.34)
	*norank_f:Desulfocapsaceae* (0.63)
	*unclassified_f:Rhodobacteraceae* (0.85)
	*Ruegeria* (0.82)
	*unclassified_c:Gammaproteobacteria* (0.60)
Late (L group)	*Catellicoccus* (39.17)
	*Methylobacterium-Methylorubrum* (5.47)
	*unclassified_c:Gammaproteobacteria* (5.21)
	*Psychrobacter* (4.23)
	*Cetobacterium* (3.96)
	*Breznakia* (3.64)
	*Sulfitobacter* (1.96)
	*norank_f:Desulfocapsaceae* (1.93)
	*Ilumatobacter* (1.90)
	*unclassified_Rhodobacteraceae* (1.83)

Stacked histograms of relative abundance at the phylum (others <1%) and genus level of the gut microbiome (others <1%) are compared in **Supplementary Figure 2**. The microbiome composition at the class, order, and family level is shown in **Supplementary Figure 3**.

### 3.3 Differences between gut microbial communities

**Figure 2:**
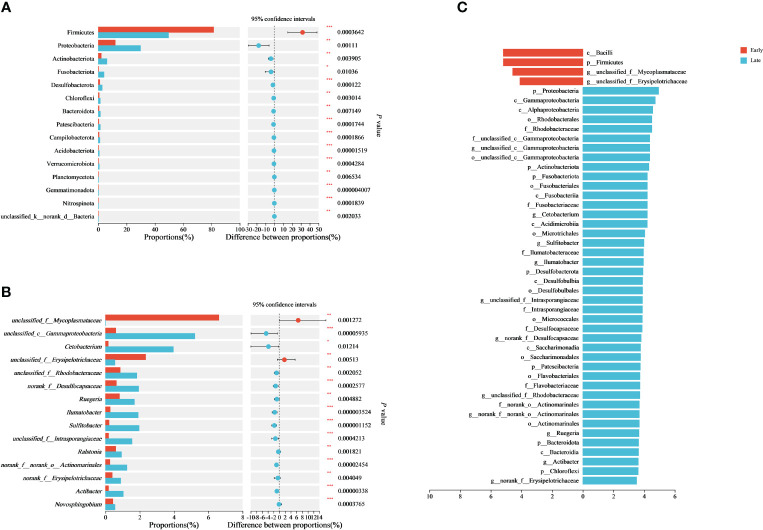
Differences in the gut microbiome and taxa identified by the LEfSe (LDA=3.5). Differential analysis of dominant bacterial phyla **(A)** and genera **(B)** between E and L groups based on Wilcoxon rank-sum tests. LEfSe analysis is based on characterizing discriminative features of OTUs **(C)**. *p < 0.05, **p < 0.01, and ***p < 0.001.

The differences in gut microbiome between the E and L groups of the relict gulls were further investigated. The Wilcoxon rank-sum test showed that the abundance of Firmicutes was significantly higher in the E group than in the L group (*p* < 0.001), whereas the abundance of Proteobacteria, Actinobacteriota, Fusobacteriota, and Desulfobacterota was significantly lower in the E group than in the L group (*p* < 0.05) (**Figure 2A**).

At the genus level, the abundance of *unclassified_f_Mycoplasmataceae* and *unclassified_f_Erysipelotrichaceae* in the E group was significantly higher than those in the L group (*p* < 0.01), whereas the abundance of *unclassified_c_Gammaproteobacteria*, *Cetobacterium*, *unclassified_f_Rhodobacteraceae*, *norank_f_Desulfocapsaceae*, *Ruegeria*, *Ilumatobacter, Sulfitobacter*, *unclassified_f_Intrasporangiaceae*, and *Ralstoria* in the E group was significantly lower than those in the L group (*p* < 0.05) (**Figure 2B**). As a result, the composition of the gut microbiome differed significantly (*p* < 0.05) between the E and L groups of relict gulls.

Using linear discriminant analysis effect size (LEfSe) analysis to examine intestinal microbial biomarkers, we identified differences in relative abundance between the E and L groups for 4 and 42 taxa (LDA = 3.5) (**Figure 2C**; **Supplementary Figure 4**). The results indicated significant differences in important characteristic biomarkers between the two groups for at the genus level (LDA = 3.5). The biomarkers for the E group were *unclassified_Mycoplasmataceae* and *unclassified_Erysipelotrichaceae* (LDA = 3.5). The biomarkers for the L group were *Erysipelotrichaceae*, *Sulfitobacter*, *unclassified_Rhodobacteraceae*, *Ruegeria*, *unclassified_Gammaproteobacteria*, *Cetobacterium*, *Actinomarinales*, *Ilumatobacter*, *unclassified_Intrasporangiaceae*, *Desulfocapsaceae*, and *Actibacter* (LDA = 3.5) (**Figure 2C**; **Supplementary Figure 4**).

### 3.4 The alpha and beta diversity of the gut microbiome from different groups

**Figure 3:**
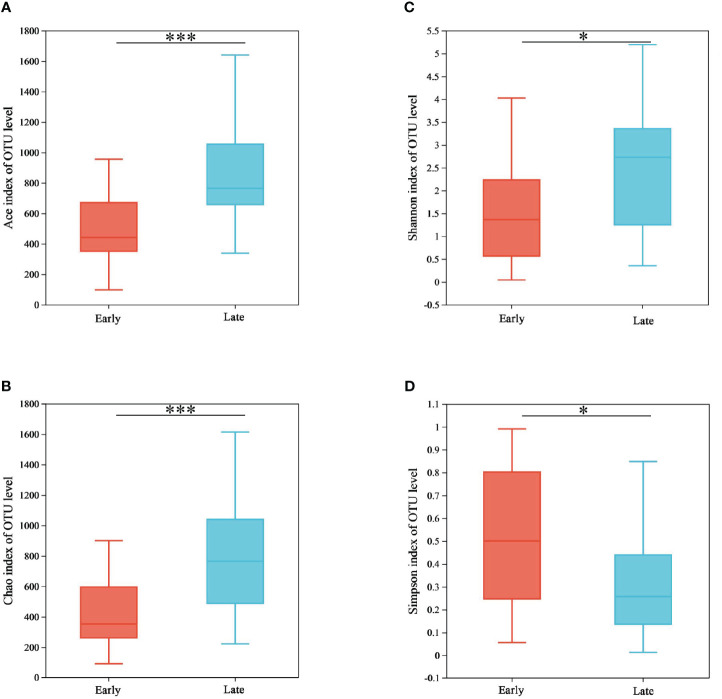
Alpha diversity between the two groups. **(A)** Bacterial community richness (ACE index), **(B)** bacterial community diversity (Shannon index), **(C)** bacterial community diversity (Chao index), and **(D)** bacterial community diversity (Simpson index). *p < 0.05 and ***p < 0.001.

For the alpha diversity analysis, the ACE, Chao1, Shannon, and Simpson indices were calculated for each sample. The SPSS 26.0 software was used to perform statistical analyses with the Wilcoxon rank-sum test, and the results revealed significant differences between the E and the L groups (*p* < 0.05) (**Figure 3**; **Supplementary Table 2**). In this study, the ACE (*p* = 0.001), Chao1 (*p* = 0.001), and Shannon (*p* = 0.014) indices were significantly higher in the L group than in the E group. In comparison, the Simpson (*p* = 0.039) index was significantly lower in the L group than in the E group. This indicates an increase in the abundance and diversity of gut microorganisms during the overwintering period in Tianjin city.

**Figure 4:**
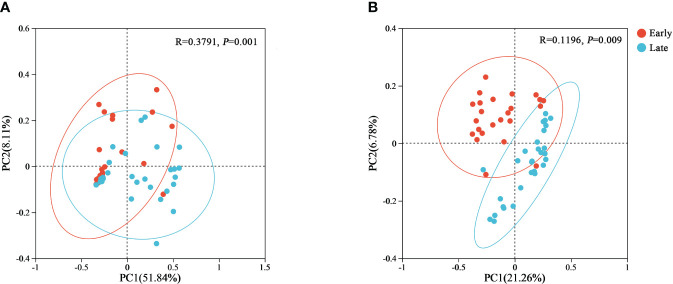
PCoA plots of beta diversity of the gut microbiome from the relict gull. PCoA plots based on **(A)** weighted and **(B)** unweighted UniFrac distances.

Beta diversity was performed using principal coordinate analysis. The PCoA plots were generated for both weighted and unweighted UniFrac metrics to determine whether differences in bacterial composition exist between different populations (**Figure 4**). The contribution of the first and second principal coordinates for the weighted UniFrac PCoA was 51.84% and 8.11%, respectively (**Figure 4A**). For the unweighted UniFrac PCoA, the contribution of the first and second principal coordinates were 21.26% and 6.78%, respectively (**Figure 4B**). The higher contributions for the weighted UniFrac also increased the reliability of the results. This analysis showed that individuals of the same group clustered together, and fecal samples from different groups were clearly separated, suggesting that there were significant differences in the bacterial composition of relict gulls between the E and L groups (weighted UniFrac, *p* = 0.001; unweighted UniFrac, *p* = 0.009) (**Figure 4**).

### 3.5 Functional predictions of the gut microbiome

**Figure 5:**
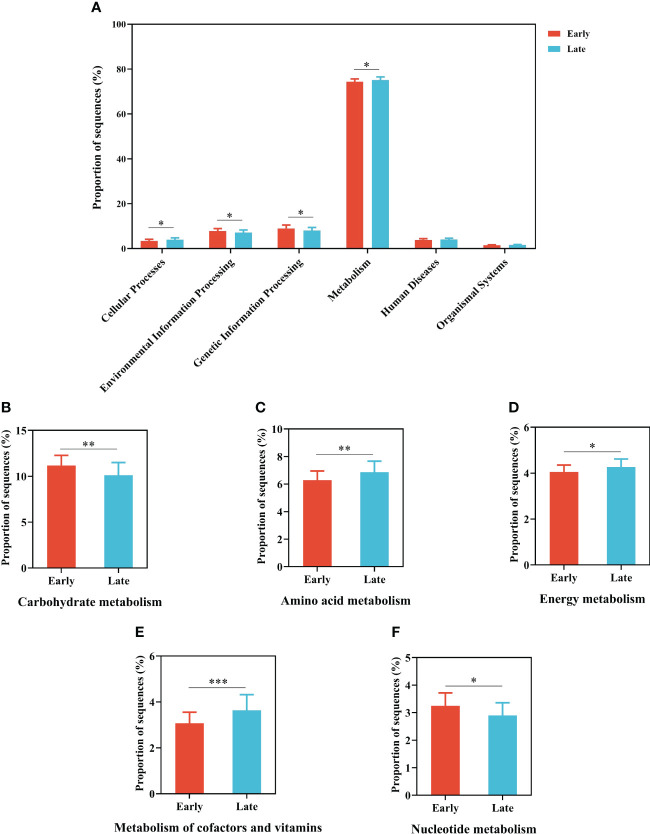
Gut microbial functional predictions of total individuals based on the KEGG database. **(A)** The results of significantly different functional predictions at KEGG level 2 between E and L groups. Relative abundance of genes associated with **(B)** carbohydrate metabolism, **(C)** amino acid metabolism, **(D)** energy metabolism, **(E)** metabolism of cofactors and vitamins, and **(F)** nucleotide metabolism predicted by PICRUSt. The error bars represent standard deviations. The difference was compared based on the *post hoc* Tukey–Kramer test. *p < 0.05, **p < 0.01, and ***p < 0.001.

The main potential functions were annotated using PICRUSt2, including metabolism, genetic information processing, environmental information processing, and cellular processes at KEGG level 1, which significantly (*p* < 0.05) differed across the two groups (**Figure 5A**). A total of 409 KEGG pathways (level 3) were classified into 46 secondary KEGG pathways (level 2) and there is significantly difference in KEGG level 2 functions between the E and L groups (**Supplementary Figures 5**).

Of these pathways, the relative abundance of metabolism accounted for more than 50% of all the pathways the whole process, and most metabolic pathways were similar. Among the latter, the relative abundance of genes related to amino acid metabolism, metabolism of cofactors and vitamins, energy metabolism, and environmental adaption in gut microorganisms in the L group was significantly higher than those in the E group (*p* < 0.05; Welch’s t-test). On the contrary, the relative abundance of genes related to carbohydrate metabolism, membrane transport, translation, and nucleotide metabolism was significantly lower (*p* < 0.05; Welch’s t-test) in the L group (i.e., when the migratory relict gull lived in the wintering area for some time) than in the E group (**Figures 5B–F**; **Supplementary Figure 5**).

In order to study the health status of the migratory birds, phenotypic predictions based on the gut microbiome composition were performed. Based on BugBase phenotypic predictions, the abundance of Potentially_Pathogenic bacteria (*p* < 0.001), Gram_Positive bacteria (*p* < 0.001), and Contains_Mobile_Elements bacteria (*p* < 0.001) were significantly higher in the E group than those associated with the L group, whereas the abundance of Forms_Biofilms bacteria (*p* < 0.001), Gram_Negative bacteria (*p* < 0.001), Aerobic bacteria (*p* < 0.001) and Stress_Tolerant bacteria (*p* < 0.01) were significantly lower in the E group than those associated with the L group (**Supplementary Figure 6**).

## 4 Discussion

The gut microbiome contributes to host health, and studying gut microorganisms will shed light on the different periods during the life cycle of endangered animals (Waite and Taylor, 2014; Zhang et al., 2021b). To the best of our knowledge, the present study is the first to investigate the gut microbiome of relict gulls during the winter, for which we concluded some important findings.

At the phylum level, the gut microbiome was dominated by Firmicutes and Proteobacteria, which have also been found to predominate among swimming birds (e.g. whooper swans (*Cygnus cygnus*): Wang et al., 2021; Baer’s pochards (*Aythya baeri*): Xi et al., 2021) and wading birds (e.g. red-crowned cranes (*Grus japonensis*): Xie et al., 2016; black-necked cranes (*Grus nigricollis*): Wang et al., 2020; hooded cranes (*Grus monacha*): Dong et al., 2019; Gu and Zhou, 2021; Zhang et al., 2021a). Furthermore, it has also been demonstrated that the fecal microbiome of Asian crested ibises (*Nipponia nippon*) (Zhu et al., 2021) and great tits (*Parus major*) (Goossens et al., 2022) is dominated by Firmicutes and Proteobacteria. Firmicutes have been found in some avian species to help the host break down complex carbohydrates, polysaccharides, and fats (Turjeman et al., 2020), thus improving the ability of hosts to absorb energy and nutrients from daily food. Proteobacteria have several physiological functions and use high-carbon sources, which help to meet the higher energy and nutritional requirements of the organism (Wang et al., 2021; Xi et al., 2021). It is worth noting that although the dominant phyla of avian species converge, there are species-specific dominant phyla, and these interspecific differences are also reflected in the diverse characteristics of the dominant genera of the species, which are related to the evolutionary status (Hoar et al., 2007), habitat environment (Berlow et al., 2021), habits (Risely et al., 2017), and food habits (Davidson et al., 2020; Zhang et al., 2021a) of the avian species. For instance, within the gull genus, the gut flora of Bradford Beach and Grant Park gulls (Koskey et al., 2014) are dominated by Firmicutes and Proteobacteria, with *Catellicoccus* as the most abundant genus, which is consistent with the results of this study. Furthermore, our results are overall in line with the dominant phylum found in wintering hooded cranes, but hooded cranes were dominated by *Clostridium* and *Epsilonproteobacteria* as the dominant genera (Dong et al., 2019), which is inconsistent with the results of this study. This suggests, in part, that the intestinal flora of species with similar evolutionary statuses converge, while the intestinal flora of species with more different evolutionary statuses vary greatly.

To date, only two related reports have been published on the gut microbiome of relict gulls. One study focused on the wild population in June (Liu et al., 2022), whereas the other focused on the captive population in May (Gao et al., 2021). The top two dominant phyla of these studies were similar to our study. Thus, gut microorganisms belonging to the phyla Firmicutes and Proteobacteria may help the relict gulls adapt to living conditions, a result consistent with previous findings in the breeding period of this species (Liu et al., 2022). At the genus level, the results of this study are consistent with those of Liu et al. (2022), with *Catellicoccus* as the dominant genus. *Catellicoccus*, belonging to Firmicutes, is widespread in various bird gut microbiomes (Ryu et al., 2014; Laviad-Shitrit et al., 2019) and is detected predominantly in gull feces (Lu et al., 2008; Koskey et al., 2014). The genome of *Catellicoccus marimammalium* shows that this bacterium encodes multiple functions (e.g., nutrient transport), suggesting that it may have beneficial effects on its hosts (Koskey et al., 2014). Furthermore, studies on thick-billed murres (*Uria lomvia*) suggested that *Catellicoccus* may help these birds to optimize their nutrition. Thus, the high abundance of *Catellicoccus* in wild relict gulls may be related to limited feeding conditions. The results of Liu et al. (2022) and Gao et al. (2021) showed that *Lactobacillus* was the co-dominant genus. Although no potential association of *Lactobacillus* has been found in avian studies, it has been shown that its abundance increases during pregnancy and lactation in humans (Koren et al., 2012). Therefore, we speculate that such differences are likely due to the different life histories of the species related to the sampling time.

Firmicutes produces short-chain fatty acid (SCFAs) that can be directly absorbed by the host’s intestinal wall as an energy source (Yadav et al., 2021). Based on the LEfSe analysis, the relative abundance of Firmicutes was significantly higher in the E group than in the L group. Therefore, a high proportion of Firmicutes may help hosts to digest food and improve energy absorption, facilitating their rapid adaptation to the overwintering environment (Zhang et al., 2021a). Erysipelotrichaceae are part of the phylum Firmicutes. Based on previous research, the levels of Erysipelotrichaceae were associated with the host’s carbohydrate consumption (Cox et al., 2013) and may have the ability to produce SCFAs (Li et al., 2020). Wu et al. (2021) also found that this strain could use plant polysaccharides. The abundance of *unclassified_Erysipelotrichaceae* belonging to the family ‘ Erysipelotrichaceae ‘ within the phylum Firmicutes was higher in the E group than in the L group, consistent with the predicted metabolic pathways, in which the carbohydrate metabolism was also higher in the E group. We speculate that the content of Firmicutes is high in the early period, while the proportion of Firmicutes decreases in the later period, mainly because the living conditions along the migration route are more diverse and involve many plants in addition to fish. After living in overwintering site Tianjin Relict Gull Park, the main food of relict gull is fish, shellfish and other marine organisms (the information was shown in **Supplementary Figures 7**, **8**). As a result, the proportion of bacteria that could help degrade plant polysaccharides in the E group is higher.

Proteobacteria have a variety of metabolic functions that facilitate the high collective requirements for energy and nutrients (Sun et al., 2022). In this study, the abundance of Proteobacteria was significantly higher in the L group than in the E group, which could be a change made by the relict gulls to adapt to cold weather and scarce food resources. This study also found that the relative abundance of Actinobacteriota was significantly higher in the L group than in the E group, consistent with the findings for overwintering of *G. nigricollis*, but its potential association with avian species has not been studied. We further found a high relative abundance of Fusobacteriota in this study, consistent with the high relative abundance of Fusobacteriota in some carnivorous birds (Waite and Taylor, 2015); therefore, we hypothesize that the high abundance of Fusobacteriota is related to the predominant consumption of high-protein foods by relict gulls during the wintering period (**Supplementary Figures 7**, **8**). *Cetobacterium*, which belongs to Fusobacteriota, is predicted to be involved in lipid metabolism (Zhu et al., 2021), and we observed a higher relative abundance of *Cetobacterium* in the L group than in the E group, improving the utilization of carbon sources after the temperature decreased in the later wintering period. The results suggest that these bacteria may play important functional roles in the relict gulls, such as food digestion, nutrient absorption, and adaptability to the environment, in agreement with previous findings on other avian species (Wang et al., 2021; Yadav et al., 2021; Sun et al., 2022).

Based on beta diversity using both weighted and unweighted UniFrac distances, the two groups were clustered separately and had significant differences. Our finding suggests that the gut microbial composition of the relict gulls varied at the early and late stages of overwintering, similar to many studies demonstrating that different periods of overwintering could have different influences on the compositional structure of microbial communities (Grond et al., 2019; Wang et al., 2021; Zhao et al., 2021) as it relates to different needs. Risely et al. (2018) found no statistically significant differences in microbial communities between migrants and residents of Calidris shorebirds based on alpha diversity (Risely et al., 2018). Inconsistent with the above finding by Risely et al. (2018), the results of this study showed that alpha diversity increased during the overwintering period and may be related to the food source and the living environment in Tianjin city (**Supplementary Figures 7**, **8**). Furthermore, through functional predictions, the proportion of sequences related to human diseases (e.g., **Figure 5**) was similar between the two groups, indicating that the pathogenic microorganisms carried by relict gulls did not change with migration and wintering grounds. Thus, our analyses of the gut microbiome of the relict gull suggest that the environment and food source for our study site are suitable for *L. relictus* to survive the winter, to some extent.

In conclusion, this study demonstrates for the first time the microbial community structure of early and late wintering relict gulls. The significant change of some predominant phyla and genera during early and late overwintering periods could be explained as an adaptation to the habitat to some extent. However, it should be mentioned that the limited sample size from one study site likely influenced the statistical power of the present study. Therefore, in future studies, a larger sample size from more habitats could be considered to further determine the comprehensive characteristics of the gut microbiome across the life history of this endangered species.

## Data availability statement

The datasets presented in this study can be found in online repositories. The names of the repository/repositories and accession number(s) can be found in the article/**Supplementary Material**.

## Author contributions

DZ, HW, ZZ and HY conceived and designed the study and wrote the manuscript. HY, ZZ, NW, MW, and QW collected the samples and conducted the experiments. DZ, HW, HY, and ZZ analyzed the data. All authors contributed to the article and approved the submitted version.
