# Supplementary material for: Comparative analysis of intestinal flora at different overwintering periods in wild relict gulls (Larus relictus): first evidence from Northern China
Source: Front Microbiomes. 2023 Oct 2;2:1218281. doi: 10.3389/frmbi.2023.1218281 (PMC12993621; doi:10.3389/frmbi.2023.1218281)
Supplement: Supplementary file 1 [file DataSheet_1.docx]

Supplementary Material

Comparative Analysis of Intestinal Flora at Different Overwintering Periods in Wild Relict Gulls (*Larus relictus*): First Evidence from Northern China

Hongyu Yao^1^†, Zeming Zhang^1^†, Nan Wu^1^, Mengping Wang^1^, Qian Wu^1^, Hong Wu^1^*, Dapeng Zhao^1^*

*** Correspondence:** Hong Wu: skywuhong@tjnu.edu.cn

Dapeng Zhao: skyzdp@tjnu.edu.cn

## Supplementary Figures


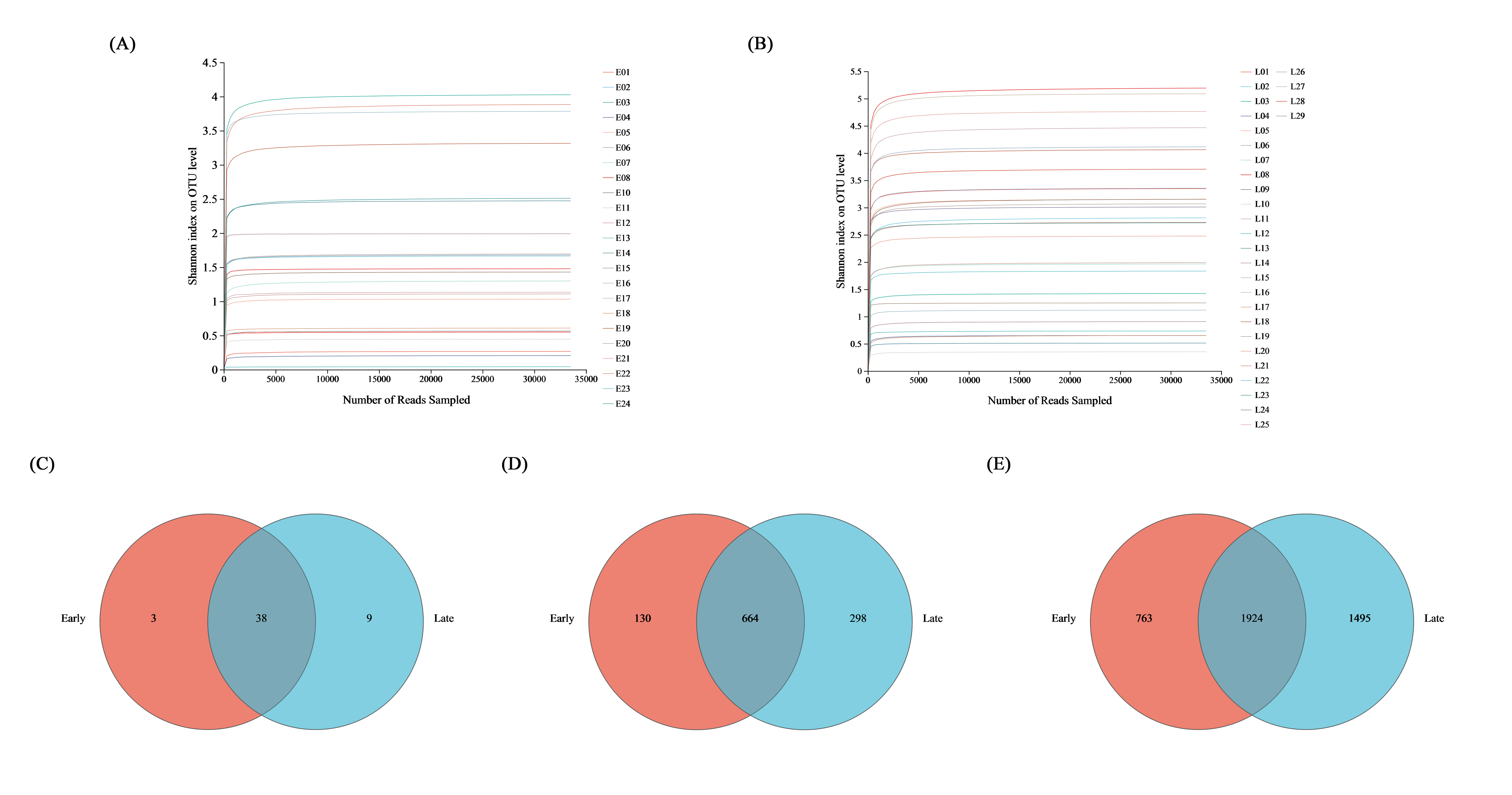


**Supplementary Figure1.** The shannon index on OTU level from E group (A) and L group (B), and the Venn diagrams at levels of Phylum (C), genus (D) and OTU (E).


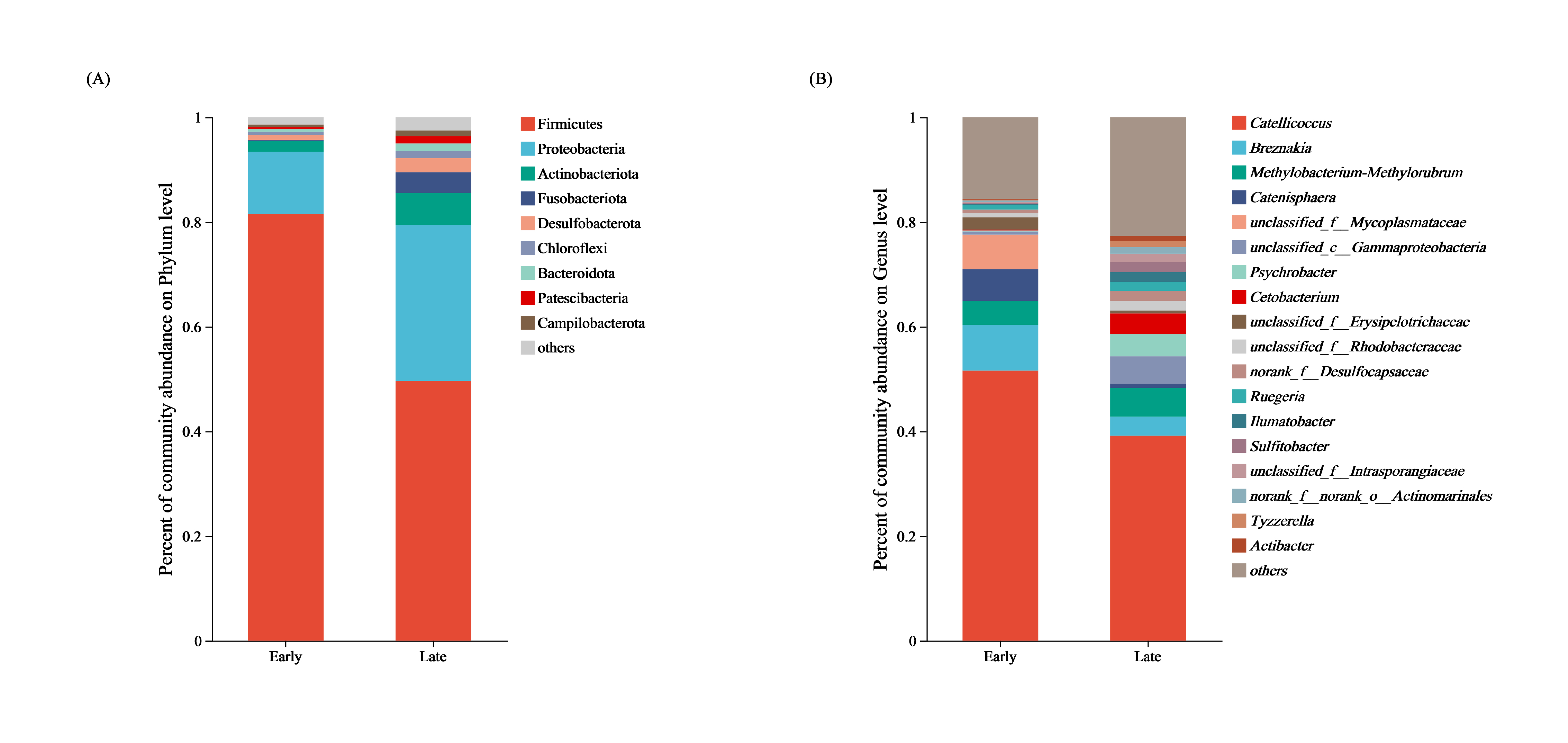


**Supplementary Figure 2.** Stacked histograms of relative abundance at the phylum level (others <1%) (A) and genus level (others <1%) (B).


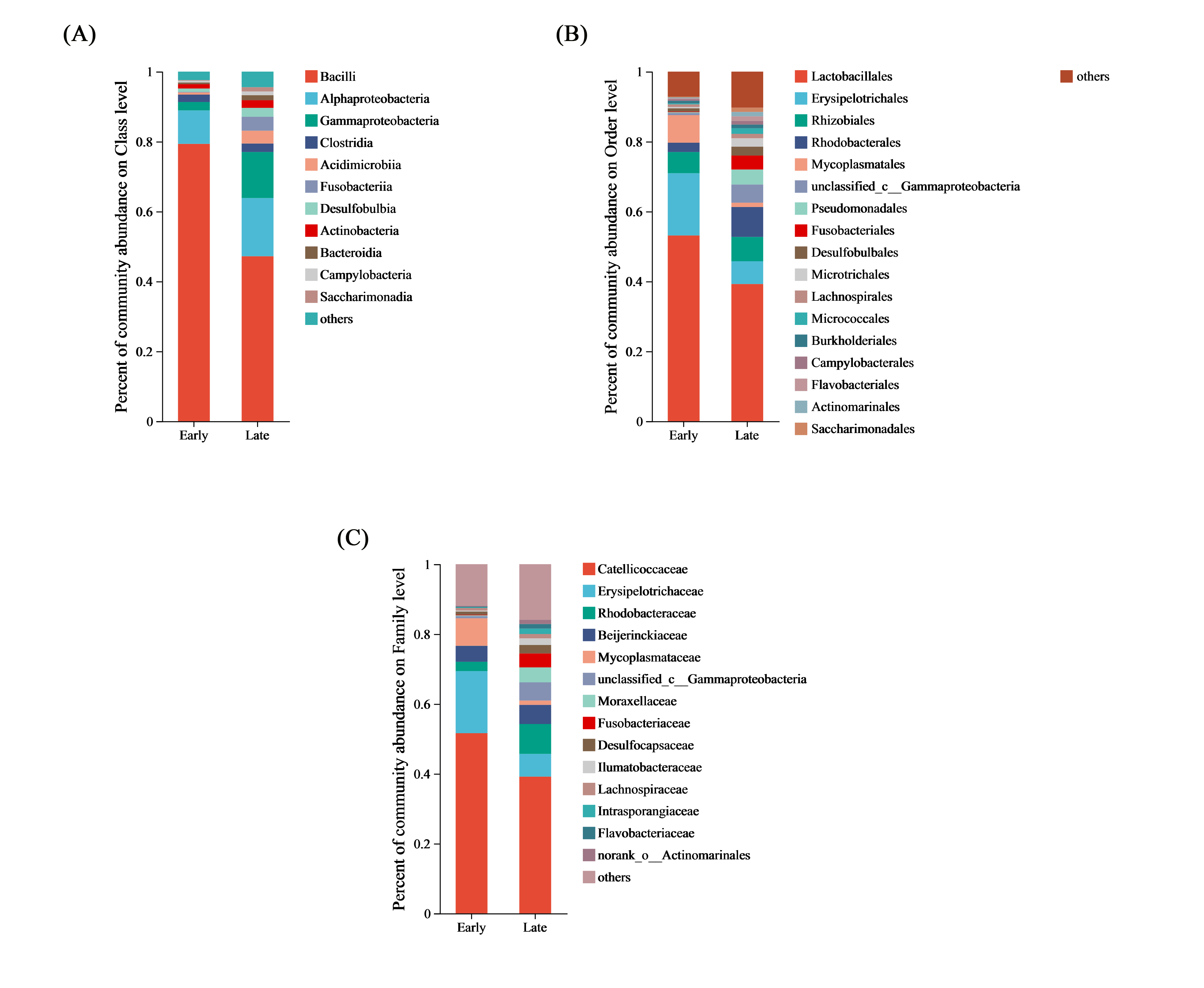


**Supplementary Figure 3.** The microbial compositions at class(A), order(B), and family levels(C) between E group and L group.


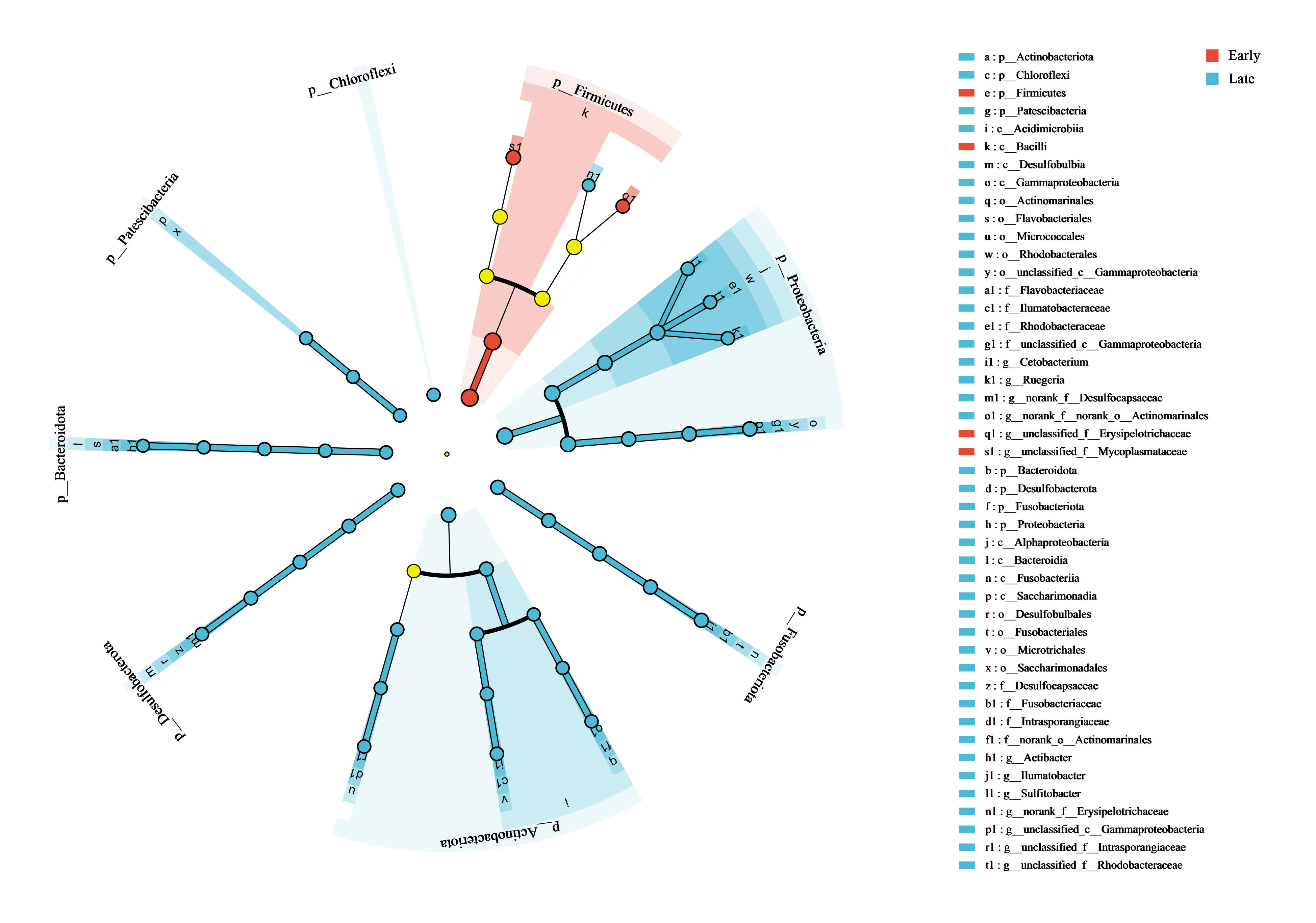


**Supplementary Figure 4.** The cladogram of the main taxa of microbiota from E group and L group by Linear discriminant analysis effect size (LEfSe) test.


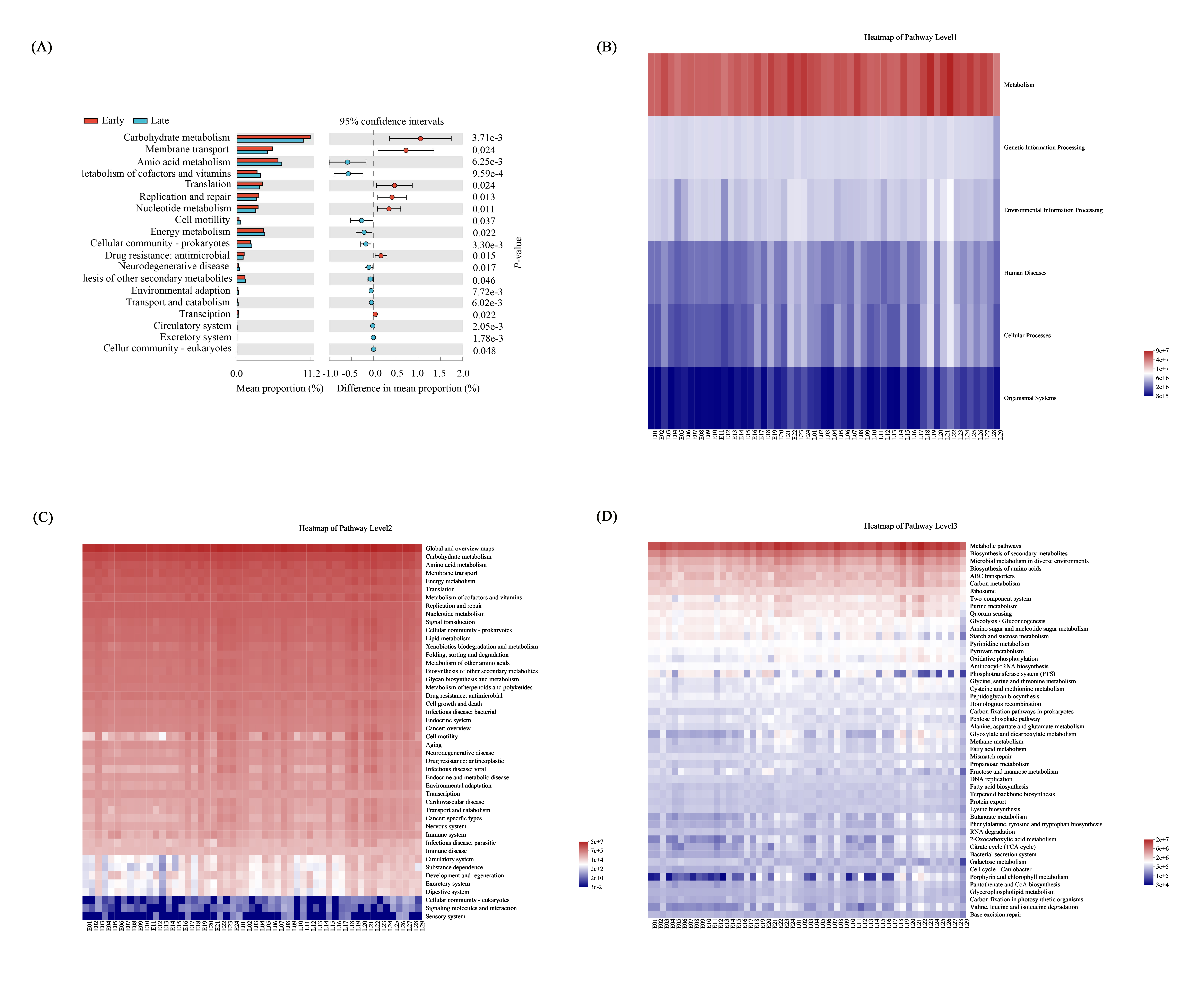


**Supplementary Figure 5.** The difference of potential functions at pathway level 2 between two groups (A) and the heatmap of main potential functions annotated using the PICRUSt2 on level 1 (B), level 2 (C) and level 3 (D).


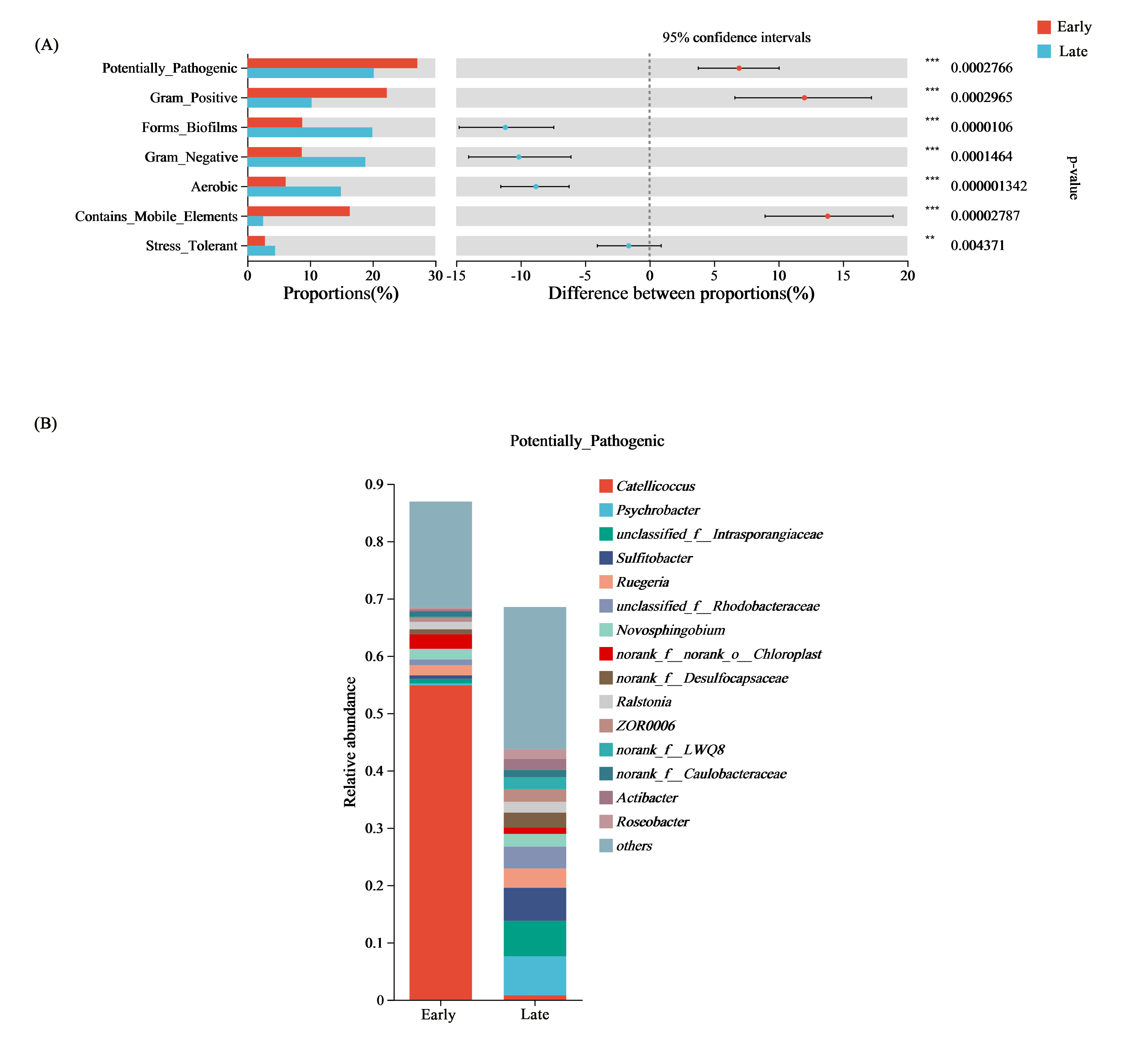


**Supplementary Figure 6.** The phenotypic prediction based on the composition of gut microbiota (A) The differences between the two groups were compared; (B) The relative abundance of the two groups.


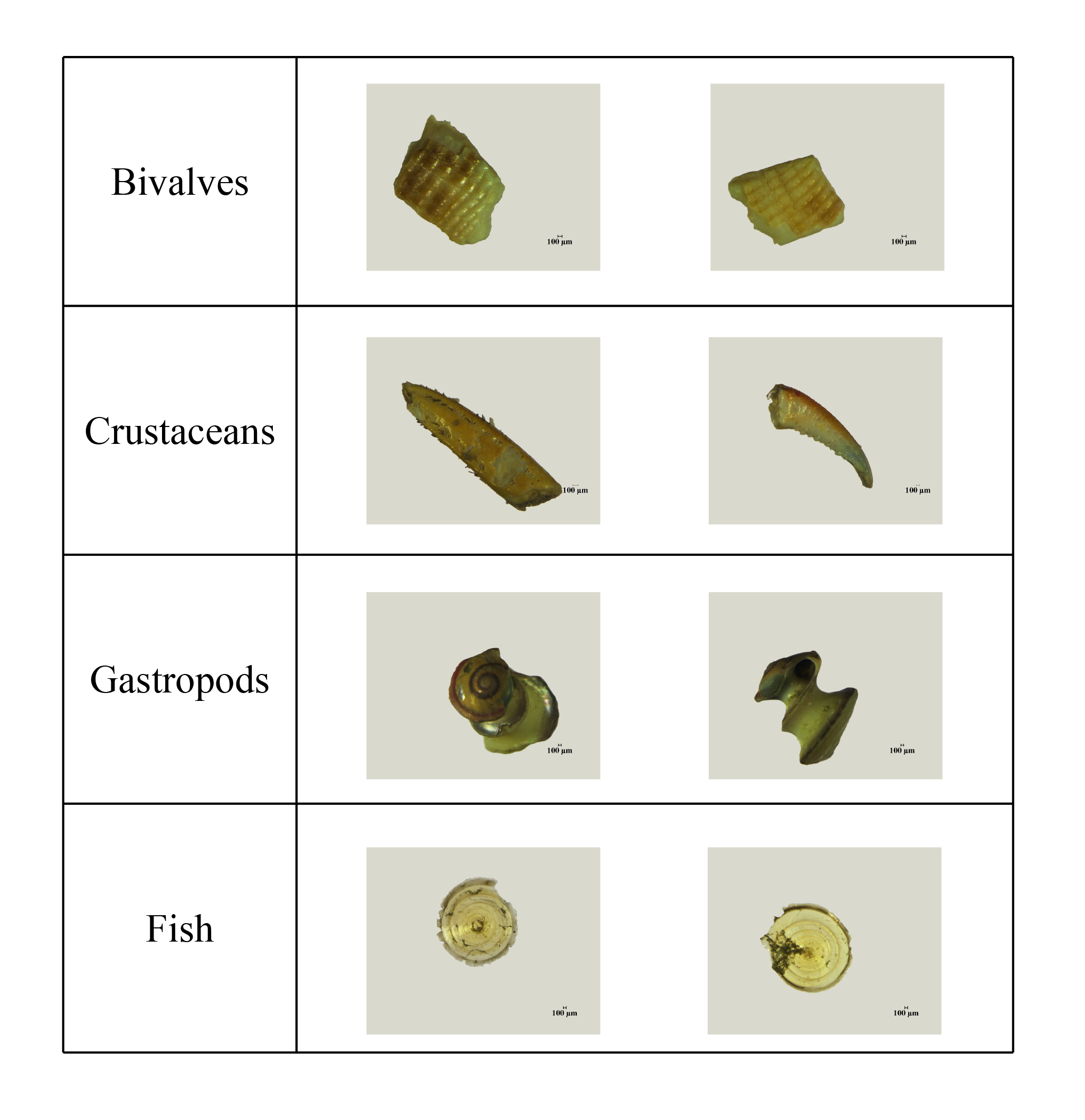


**Supplementary Figure 7.** The residue of bivalves, crustaceans, gastropods and fish found in fecal samples from E group by fecal microscopy.


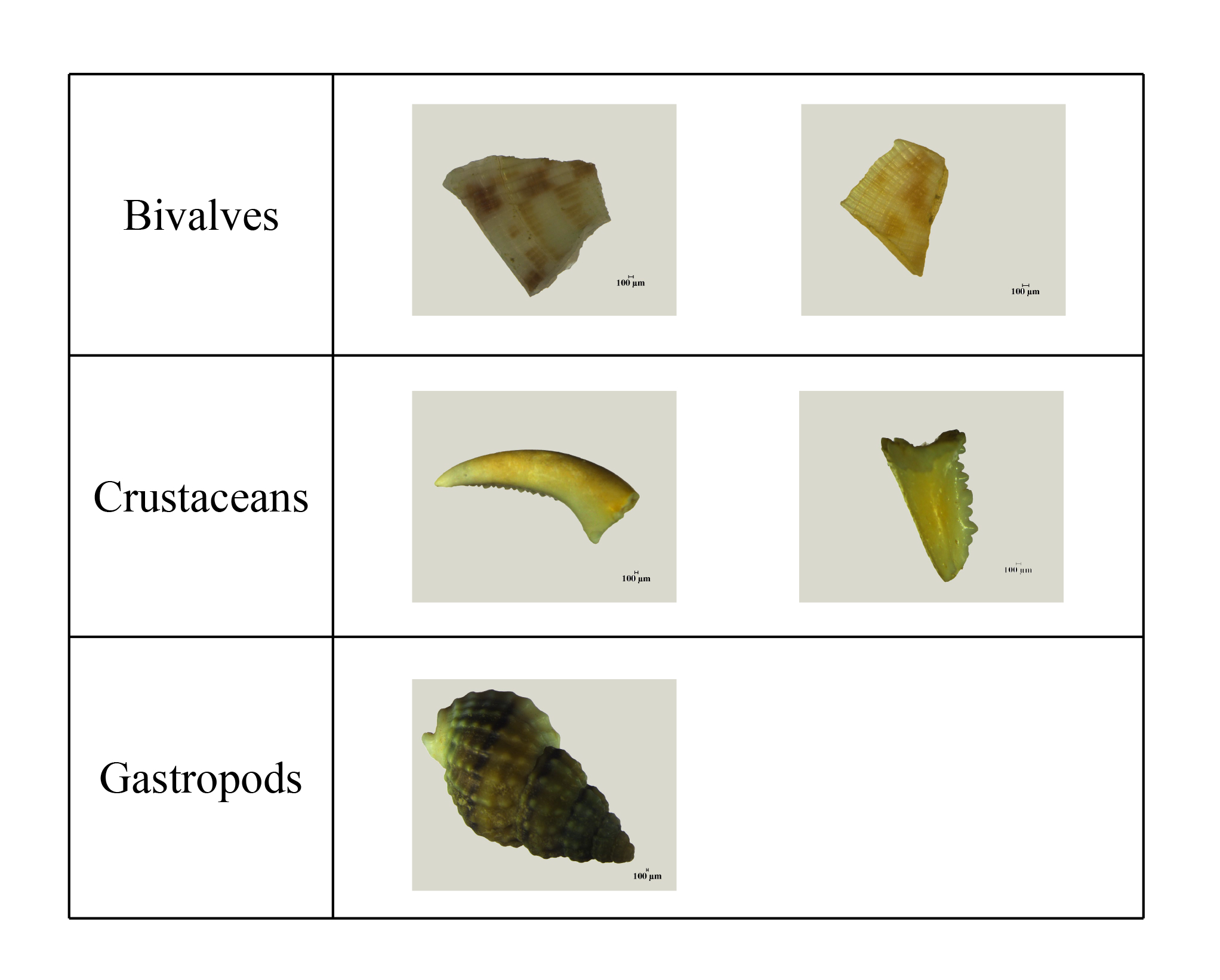


**Supplementary Figure 8.** The residue of bivalves, crustaceans, and gastropods found in fecal samples from L group by fecal microscopy.

## Supplementary Tables

**Table S1.** The total number of raw reads, base pairs, the mean length of the reads.

| **Sample** | **Seq_num** | **Base_num** | **Mean_length** | **Min_length** | **Max_length** |
| --- | --- | --- | --- | --- | --- |
| E01 | 42334 | 18138161 | 428.453749 | 274 | 504 |
| E02 | 59323 | 25432923 | 428.719434 | 352 | 431 |
| E03 | 46651 | 19533994 | 418.726158 | 270 | 475 |
| E04 | 35814 | 15354345 | 428.724661 | 340 | 431 |
| E05 | 39360 | 16836439 | 427.755056 | 402 | 460 |
| E06 | 39777 | 16945519 | 426.012997 | 270 | 452 |
| E07 | 39989 | 17071971 | 426.916677 | 213 | 511 |
| E08 | 44823 | 19215976 | 428.70794 | 374 | 431 |
| E09 | 47288 | 20156567 | 426.251205 | 262 | 430 |
| E10 | 38185 | 16334562 | 427.774309 | 277 | 431 |
| E11 | 40568 | 17378407 | 428.377218 | 219 | 432 |
| E12 | 39672 | 16988972 | 428.235834 | 277 | 431 |
| E13 | 40225 | 17252620 | 428.902921 | 277 | 439 |
| E14 | 40702 | 17243610 | 423.655103 | 219 | 475 |
| E15 | 44981 | 19231055 | 427.537294 | 277 | 539 |
| E16 | 37102 | 15863582 | 427.566762 | 224 | 432 |
| E17 | 55358 | 23318488 | 421.230680 | 347 | 443 |
| E18 | 41641 | 17855209 | 428.789150 | 369 | 432 |
| E19 | 55776 | 23076573 | 413.736607 | 364 | 460 |
| E20 | 37694 | 16044654 | 425.655383 | 262 | 453 |
| E21 | 42872 | 18299484 | 426.839989 | 402 | 430 |
| E22 | 58654 | 24167662 | 412.037747 | 283 | 516 |
| E23 | 40657 | 17162004 | 422.116831 | 374 | 432 |
| E24 | 39174 | 16218072 | 414.000919 | 337 | 505 |
| L01 | 51197 | 21369254 | 417.392699 | 375 | 431 |
| L02 | 48628 | 20535482 | 422.297483 | 262 | 432 |
| L03 | 49633 | 21221322 | 427.564765 | 374 | 432 |
| L04 | 46624 | 19953243 | 427.960771 | 374 | 488 |
| L05 | 48811 | 20630146 | 422.653623 | 244 | 431 |
| L06 | 52500 | 22050795 | 420.015143 | 373 | 455 |
| L07 | 50157 | 21377359 | 426.208884 | 265 | 516 |
| L08 | 51866 | 21520378 | 414.922647 | 262 | 432 |
| L09 | 50171 | 21186064 | 422.277092 | 259 | 459 |
| L10 | 51619 | 22124716 | 428.615742 | 352 | 431 |
| L11 | 51841 | 22071301 | 425.749908 | 374 | 455 |
| L12 | 86867 | 35573798 | 409.520278 | 262 | 438 |
| L13 | 48944 | 20953480 | 428.111311 | 384 | 452 |
| L14 | 52423 | 22396388 | 427.224463 | 375 | 484 |
| L15 | 48705 | 20314112 | 417.084735 | 239 | 478 |
| L16 | 49749 | 21288529 | 427.918732 | 346 | 477 |
| L17 | 47714 | 20405589 | 427.664606 | 302 | 432 |
| L18 | 40904 | 16969962 | 414.872922 | 253 | 464 |
| L19 | 58272 | 23937880 | 410.795579 | 364 | 516 |
| L20 | 54078 | 23043585 | 426.117552 | 374 | 432 |
| L21 | 38521 | 15986226 | 415.000286 | 374 | 437 |
| L22 | 58308 | 23961024 | 410.938876 | 373 | 432 |
| L23 | 40843 | 17151627 | 419.940430 | 374 | 431 |
| L24 | 38797 | 16197378 | 417.490476 | 367 | 431 |
| L25 | 54289 | 22376343 | 412.170845 | 364 | 455 |
| L26 | 34372 | 14430752 | 419.840335 | 364 | 432 |
| L27 | 40426 | 16785333 | 415.211324 | 374 | 525 |
| L28 | 45371 | 19101172 | 420.999581 | 364 | 481 |
| L29 | 37289 | 15986409 | 428.716485 | 207 | 431 |

**Table S2.** Estimated OTU richness and diversity indexes for each fecal sample.

| Sample ID | ACE | Chao | Shannon | Simpson | Coverage |
| --- | --- | --- | --- | --- | --- |
| E01 | 357.4607 | 275.2500 | 0.267067 | 0.937852 | 0.997553 |
| E02 | 442.7727 | 292.4762 | 0.566386 | 0.806944 | 0.997613 |
| E03 | 1189.145 | 1193.869 | 4.026585 | 0.122952 | 0.992332 |
| E04 | 603.1913 | 323.2609 | 0.205036 | 0.951188 | 0.997344 |
| E05 | 360.9274 | 292.1622 | 1.032132 | 0.670179 | 0.997673 |
| E06 | 444.5798 | 401.6389 | 1.679502 | 0.339475 | 0.996539 |
| E07 | 738.8163 | 617.3971 | 1.298541 | 0.617719 | 0.995047 |
| E08 | 397.4048 | 263.1667 | 0.549629 | 0.796958 | 0.997911 |
| E09 | 340.4573 | 259.7500 | 1.477091 | 0.413621 | 0.997941 |
| E10 | 573.5055 | 433.5250 | 1.429121 | 0.363036 | 0.996449 |
| E11 | 176.2609 | 152.7778 | 0.445434 | 0.850564 | 0.998568 |
| E12 | 472.8268 | 404.7000 | 1.108477 | 0.586807 | 0.996957 |
| E13 | 411.0659 | 186.2500 | 0.042317 | 0.990597 | 0.998986 |
| E14 | 588.4815 | 574.3659 | 2.472148 | 0.273888 | 0.995793 |
| E15 | 97.58263 | 89.10000 | 1.989581 | 0.212813 | 0.999344 |
| E16 | 342.4124 | 334.3488 | 1.133249 | 0.611428 | 0.997255 |
| E17 | 789.3920 | 776.9126 | 3.783535 | 0.055614 | 0.994570 |
| E18 | 257.1668 | 224.6667 | 0.608470 | 0.739946 | 0.997852 |
| E19 | 919.2249 | 895.6735 | 3.314775 | 0.101573 | 0.994092 |
| E20 | 439.0309 | 430.3182 | 1.694472 | 0.399495 | 0.996837 |
| E21 | 343.5415 | 196.0000 | 0.562849 | 0.813046 | 0.998299 |
| E22 | 1194.737 | 1183.768 | 3.884202 | 0.099438 | 0.993108 |
| E23 | 381.2155 | 370.1961 | 1.664468 | 0.350383 | 0.997076 |
| E24 | 955.9941 | 900.1875 | 2.509654 | 0.220112 | 0.992750 |
| L01 | 764.6629 | 765.1845 | 3.346354 | 0.135815 | 0.995614 |
| L02 | 1640.372 | 1228.273 | 2.810378 | 0.307852 | 0.989915 |
| L03 | 705.8952 | 560.3333 | 1.421993 | 0.438641 | 0.995435 |
| L04 | 362.7822 | 343.0612 | 0.651053 | 0.811525 | 0.996986 |
| L05 | 1053.967 | 1076.028 | 3.152268 | 0.198972 | 0.992153 |
| L06 | 1118.171 | 1080.439 | 3.354615 | 0.159272 | 0.991526 |
| L07 | 721.4081 | 722.7838 | 1.964113 | 0.401208 | 0.994271 |
| L08 | 1651.917 | 1613.247 | 5.192281 | 0.013035 | 0.988572 |
| L09 | 1460.413 | 1391.695 | 3.152358 | 0.228620 | 0.988692 |
| L10 | 503.8538 | 313.0323 | 0.352743 | 0.896118 | 0.997255 |
| L11 | 1189.425 | 936.8738 | 1.989615 | 0.386686 | 0.992302 |
| L12 | 477.5091 | 305.6818 | 0.733652 | 0.741774 | 0.997523 |
| L13 | 507.9380 | 367.0435 | 0.512121 | 0.848156 | 0.997195 |
| L14 | 742.2682 | 450.5682 | 0.906334 | 0.696585 | 0.996121 |
| L15 | 1511.925 | 1427.727 | 4.466005 | 0.045616 | 0.989259 |
| L16 | 732.8253 | 472.3333 | 1.116936 | 0.566022 | 0.996091 |
| L17 | 550.2047 | 488.2727 | 0.650751 | 0.838306 | 0.995584 |
| L18 | 942.9046 | 905.0408 | 4.061109 | 0.047639 | 0.993824 |
| L19 | 962.3168 | 945.4524 | 3.067454 | 0.257411 | 0.993346 |
| L20 | 657.5816 | 686.3971 | 2.475803 | 0.195277 | 0.995047 |
| L21 | 850.5618 | 845.9744 | 2.728393 | 0.180138 | 0.993913 |
| L22 | 483.6862 | 494.7143 | 1.833322 | 0.409670 | 0.996420 |
| L23 | 737.6767 | 708.8812 | 2.719622 | 0.258684 | 0.994540 |
| L2 | 741.5000 | 749.6395 | 3.009911 | 0.145040 | 0.994540 |
| L25 | 1037.701 | 1039.508 | 4.763051 | 0.026331 | 0.994391 |
| L26 | 938.5531 | 914.2214 | 4.113907 | 0.054694 | 0.994420 |
| L27 | 1093.953 | 1098.123 | 5.088339 | 0.014112 | 0.994868 |
| L28 | 983.9611 | 982.0484 | 3.703183 | 0.106929 | 0.993406 |
| L29 | 338.3795 | 222 | 1.249795 | 0.406527 | 0.99809 |
